# Determination of reasonable ice amount in foam box of pasteurized milk under air logistics mode

**DOI:** 10.1371/journal.pone.0310798

**Published:** 2024-10-31

**Authors:** Shemiao Feng

**Affiliations:** Management School, Guangzhou Civil Aviation College, Guangzhou, Guangdong, China; Bowen University, NIGERIA

## Abstract

To determine the relationship between the ice amount of packing for transportation of pasteurized milk and the temperature in the foam box and the transportation time under a certain external temperature, and to obtain the appropriate ice amount data. Considering the influencing factors of foam box ice aviation logistics and the actual operation mode, the energy consumption model of foam box ice transportation was established, and the energy consumption balance process was analyzed. On this basis, the ice amount of foam box ice transportation was set as different standards, and the corresponding relationship between the logistics duration and the temperature in the foam box was studied under different external temperatures and 50%~20% ice amount. The relationship curve between the ice proportion and the low temperature time in the foam box, the arrival time of the lowest temperature in the box, and the transportation time under the appropriate temperature was made. Research suggests that the ice amount of 50% can meet all the usual external temperatures within 60 hours of transportation quality. The logistics duration is less than or equal to 24 hours, the recommended ice amount is about 20%. The logistics duration is about 36 hours, the recommended ice amount is about 30%. The logistics duration is about 48 hours, the recommended ice amount is not more than 40%.

## Introduction

With the evolution of China’s industrial structure and the upgrading of consumption structure, the proportion of fresh goods in the transportation and logistics industry has increased year by year. As a result, there is a growing demand for cold chain logistics. the demand for cold chain logistics has steadily increased [1]. Without considering the impact of the COVID-19 epidemic, data show that nearly 1.3 billion tons of fresh products consumed in China in 2019 [2], of which about 60% need to use cold chain logistics, but according to the survey of government departments, the proportion of cold chain transportation in actual circulation is less than 20%. The main reason for this situation is the high cost of cold chain logistics in the strict sense, which requires strong consumption power and a higher level of economic development to support [3]. Therefore, at present, in addition to a few demanding fresh goods in China using cold chain transportation, the transportation of general fresh goods mainly adopts fresh storage transportation, mainly including mechanical cold storage transport, cold plate cold storage transport, liquid nitrogen cold storage transport and other ways [4].

The above several modes of fresh-keeping transport cost is higher, and the operation is more professional and complex, so the scope of use is not popular, and the foam box with ice is the most widely used refrigerated transport mode, especially the e-commerce logistics mode is widely used [5]. This study takes the major e-commerce platforms in China (Taobao, Jingdong, Pinduoduo) as an example, and finds that the vast majority of merchants adopt the foam box with ice logistics model. The reason why foam box with ice is widely used is that it is cost-effective and can greatly reduce transportation costs while maintaining appropriate transportation quality [6]. To transport fresh milk with foam box and ice, for example, from the origin of Inner Mongolia to South China, the transport cost per kilogram is only a few yuan, and the strict sense of air cold chain express, in addition to the packaging fee, the freight cost per kilogram is more than 20 yuan [7]. However, the foam box with ice mode also has disadvantages, because its temperature control ability is poor, the refrigeration temperature is unstable, and it is greatly affected by the external environment [8]. From the perspective of ensuring the quality of transportation, the greater the amount of ice added, the better the quality of transportation, but from the perspective of commercial operations, in the case of a certain foam box capacity, the greater the amount of ice added, the effective load of goods will be reduced, and the transportation cost per unit of goods will rise, so according to the transportation time and transportation environment, under the premise of ensuring customer satisfaction with the quality of logistics, it is important to determine the appropriate amount of ice added. According to the author’s survey of the e-commerce industry, at present, in actual operation, most practitioners are based on experience to estimate the amount of ice, which has a great deal of randomness and blindness [9, 10]. If the amount of ice is too little, it will cause hidden dangers to ensure the transportation quality of pasteurized milk, and if the amount of ice is too much, it will cause unnecessary losses. The reason why this situation exists is that there is a lack of in-depth research on this issue, so there is no more accurate data on the amount of ice added [11]. Only a limited number of papers can be found in common databases by searching with keywords such as "foam box" and "amount of ice added". Therefore, this paper carried out a study on this problem, the purpose is to more accurately determine the relationship between the different amount of ice, the temperature in the foam box, and the transportation time under a certain external environment temperature, and get the appropriate amount of ice.

## Materials and methods

### Materials and reagents

Pasteurized milk, packaged in 450ml bags, is a brand of sterilized fresh milk. Ice packs (food grade PE+PET double compound) of 250ml capacity were also used. There are more types of foam boxes. polystyrene foam boxes (commonly known as EPS boxes, polystyrene Expanded Polystyrene) are widely used in refrigerated transportation in China, and the closed cavity structure distributed in EPS makes it have good heat insulation and very low heat conductivity [12]. In this study, an EPS foam box of 510×300×260mm was taken as an example, with a surface area of 0.73㎡, a dead weight of 0.187kg, a thermal conductivity of 0.036W/(m·K), and a specific heat of 1.5 kJ/kg·°C. The specific heat of pasteurized milk was calculated according to 2.5×10^3^J/(kg·°C).

### Instruments and equipment

In order to simulate the external environment of the ice-adding air transport logistics process, air conditioning equipment (Gree KFR-50LW) is set in a relatively closed experimental space, and the external temperature that needs to be simulated is set on time. Set a temperature measuring device (SIGNAL WST-491-SGN) in the foam box to observe and record the temperature in the foam box regularly.

### Experimental methods

#### Energy consumption model during the transportation of foam box with ice

In the logistics mode of foam box with ice, pasteurized milk, ice and foam box constitute a sealed package. In the logistics process, there are five cold consumption items, namely heat transfer cold consumption (*Q*_1_), heat leakage cold consumption (*Q*_2_), solar radiation cold consumption (*Q*_3_), goods cooling cold consumption (*Q*_4_), box cooling cold consumption (*Q*_5_), unit: kJ. The calculation formula of each cooling consumption is as follows:

$$Q_{1}=F_{box}K_{box}(t_{out}-t_{in})Z$$ (1)

$$Q_{2}=0.1Q_{1}$$ (2)

$$Q_{3}=\gamma F_{box}K_{box}(t_{shine}-t_{out})Z_{shine}$$ (3)

$$Q_{4}=m_{c\arg o}c_{c\arg o}\Delta t$$ (4)

$$Q_{5}=m_{box}c_{box}(t_{initial}\frac{t{'}_{out}+t{'}_{in}}{2})$$ (5)

The meanings of the above symbols are as follows: *F*_*box*_ represents the outer area of the foam box (m^2^), *K*_*box*_ represents the heat transfer coefficient of the foam box (W/(m^2^⋅K)), *t*_*out*_ represents the average temperature outside the foam box (°C), *t*_*in*_ represents the temperature inside the foam box (°C), *Z* represents the heat transfer time (h), 0.1 is the heat leakage coefficient under normal circumstances. *γ* represents the percentage of the total area of the foam box that is irradiated by the sun (%), *t*_shine_ represents the temperature of the surface of the foam box that is irradiated by the sun (°C), *Z*_shine_ represents the time that the sun irradiated the foam box during the logistics process (h), *m*_box_ represents the weight of the box that needs to be cooled (kg). *c*_box_ represents the specific heat of the box that needs to be cooled (kJ/kg°C), *t*_*initial*_ represents the initial temperature of the foam box (°C), *m*_*c*arg*o*_ represents the weight of the goods in the foam box (kg), *c*_*c*arg*o*_ represents the specific heat of the goods (kJ/kg°C), and Δ*t* represents the degree of cooling down of the goods during logistics(°C). *t*'_*out*_ represents the air temperature outside the box at the end of the calculation period (°C) and *t*'_*in*_ represents the air temperature inside the box at the end of the calculation period (°C).

The above is the sum of the cold consumption, to offset this cold consumption is the cold source, in the foam box and ice packaging mode, is the ice cooling capacity. When the heat source and cold source are equal, the temperature in the foam box can reach or maintain the required level. When the cooling capacity of the ice is greater than the heat transfer of the outside world to the foam box (*Q*_1_+*Q*_2_+*Q*_3_) and the sum of the cooling consumption of the goods and the cooling consumption of the box (*Q*_4_+*Q*_5_), the remaining part is used to cool the goods and the foam box. The more cooling capacity there is left, the more cooling there is, and there is no cooling without surplus. The cooling amount is less than the sum of heat, and the temperature in the box should be gradually increased.

Taking into account most of the actual situation at present, the pasteurized milk and foam packaging boxes have been pre-cooled before entering the logistics, strictly sealed according to the requirements, and generally will not be deliberately exposed to the sun for a long time, so *Q*_2_ and *Q*_3_ are not considered, then the total cold consumption during the transportation process equal to *Q*_1_+*Q*_4_+*Q*_5_.

The cold source of the foam box is the cold amount released by the melting of the ice and salt mixture in the box, which is expressed by *Q*_0_:

$$Q_{0}=\lambda_{ice}m_{ice}+c_{ice}m_{ice}(0‐t_{ice-initial})+c_{water}m_{ice}(t_{in}-0)$$ (6)

In the formula, *λ*_ice_ represents the solubility heat of ice (kJ/kg°C), *c*_*ice*_ represents the specific heat of ice (kJ/kg°C), *c*_*water*_ represents the specific heat of water (kJ/kg°C), *m*_*ice*_ represents the amount of ice added in the foam box (kg), *t*_*ice−initial*_ represents the initial temperature of ice (°C). *t*_*in*_ stands for temperature (°C) in the foam box.

By combining the Formulas (1), (4), (5) and (6), the relationship between the amount of ice added, the transportation time, the temperature in the box and the external temperature can be obtained.

#### Setting of transportation time, ambient temperature and ice addition amount

In the e-commerce logistics mode, the operation method of the foam box with ice preservation and transportation of pasteurized milk is to evenly place the ice pack in the foam box, and then put the pre-cooled packaged pasteurized milk into the box, and then add the heat insulation pad on the top and surrounding of the goods, and pour a small amount of ice water on the heat insulation pad, and finally add the cover and wrap with sealing glue to completely block the gap and form a complete package [13].

The operation process is as follows: generally, the order is obtained on the first day, the goods are prepared, the goods are distributed to the airport on the second day, the goods are transported to the destination airport on the same day or the third day, and the goods are distributed to the customer on the third day. It normally takes no more than 48 hours for the pasteurized milk to leave the seller’s cold storage until the customer receives the goods. In this study, considering the extreme case, the logistics process is simulated according to 60 hours [14, 15].

The whole air logistics process can be divided into three periods: before installation, air transport process, and destination distribution.

1. The period before installation refers to the time when the pasteurized milk leaves the seller’s warehouse, is packaged and distributed to the airport for delivery to the carrier for temporary storage [16]. This period of time is generally determined with the flight time. Considering the abnormal circumstances such as flight delay, the extreme value of 18 hours is taken for simulation test in this study, and the ambient temperature during this period is set according to the natural external temperature.
2. The process of air transport means after the carrier receives the aircraft until it lands at the airport of destination; This period is divided into two processes. One is the temporary storage before loading into the cargo hold of the aircraft and after landing at the destination airport. The test environment temperature is simulated according to the natural external temperature, and the duration is set according to 12 hours. The second is the temperature setting during the flight [17]. China’s air transport, whether it is professional aviation cold chain logistics enterprises (such as SF Express) or airlines, is basically based on narrow-body aircraft. Take the popular A320 as an example, the CARGO HEATING system can keep the temperature of the cargo hold between 5 and 26°C, and the adjustment knob of the cockpit is placed at 12 o ’clock by default, when the temperature of the cargo hold is about 16°C, so the test ambient temperature is set to 16°C and the duration is set according to 6h.
3. Destination ground delivery refers to the delivery of pasteurized milk from the airport to the consignee. The test environment temperature during this period is set according to the natural external temperature; The duration of this simulation is set at 24 hours to account for anomalies such as transit processes, delayed or busy deliveries at the distribution center [18, 19].

In order to simplify the calculation, the amount of ice added is set to different proportions of 20%, 30%, 40% and 50%. Where, the proportion of ice added equal to ice mass divided by pasteurized milk mass and ice mass.

### Data processing

The measured data was calculated by computer programming, and the Origin 2018b 64Bit version was used.

## Results and analysis

### Relationship between logistics duration, external ambient temperature and temperature in the box

**Fig 1:**
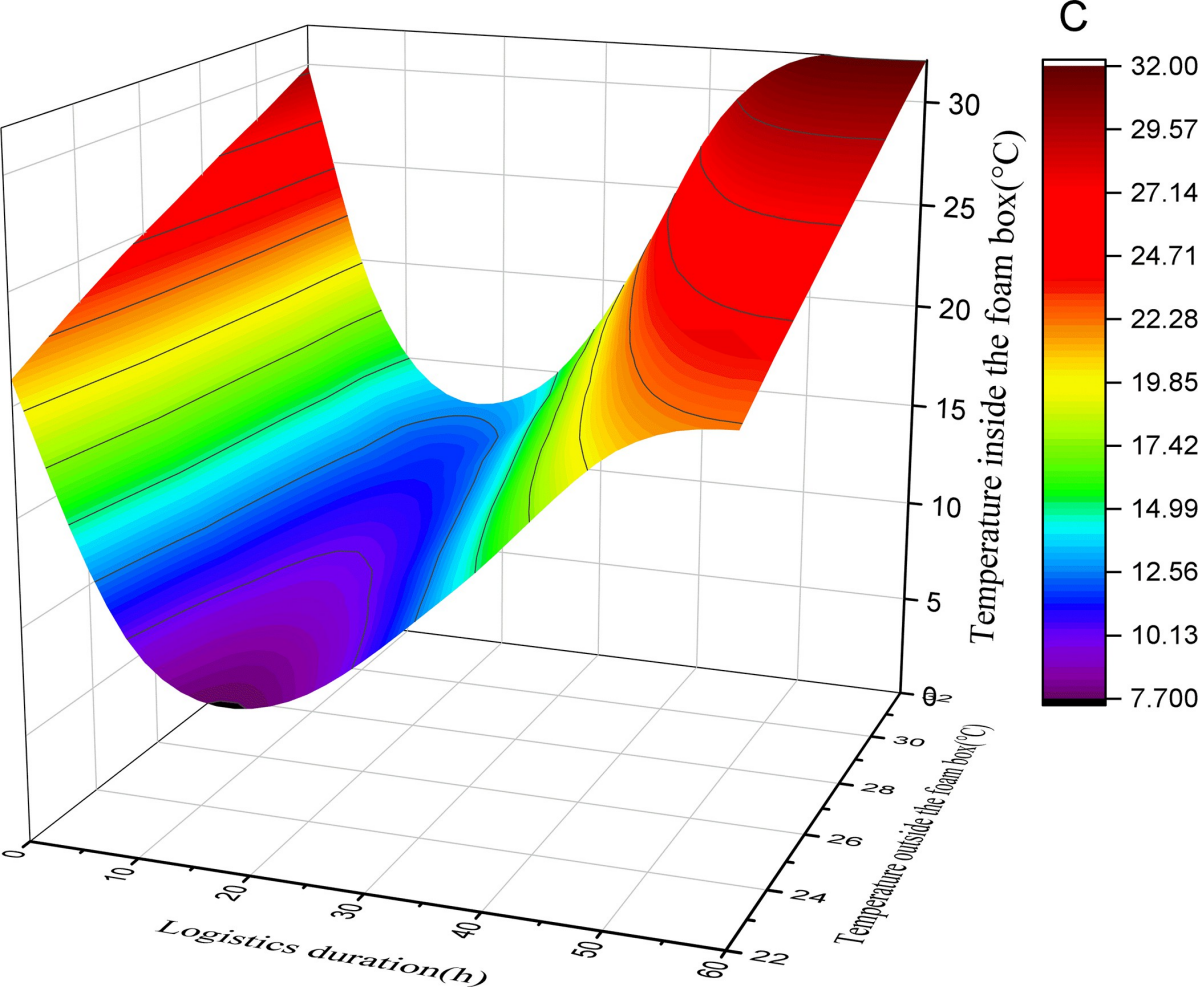
Surface diagram of temperature change in foam box (20% with ice).

**Fig 2:**
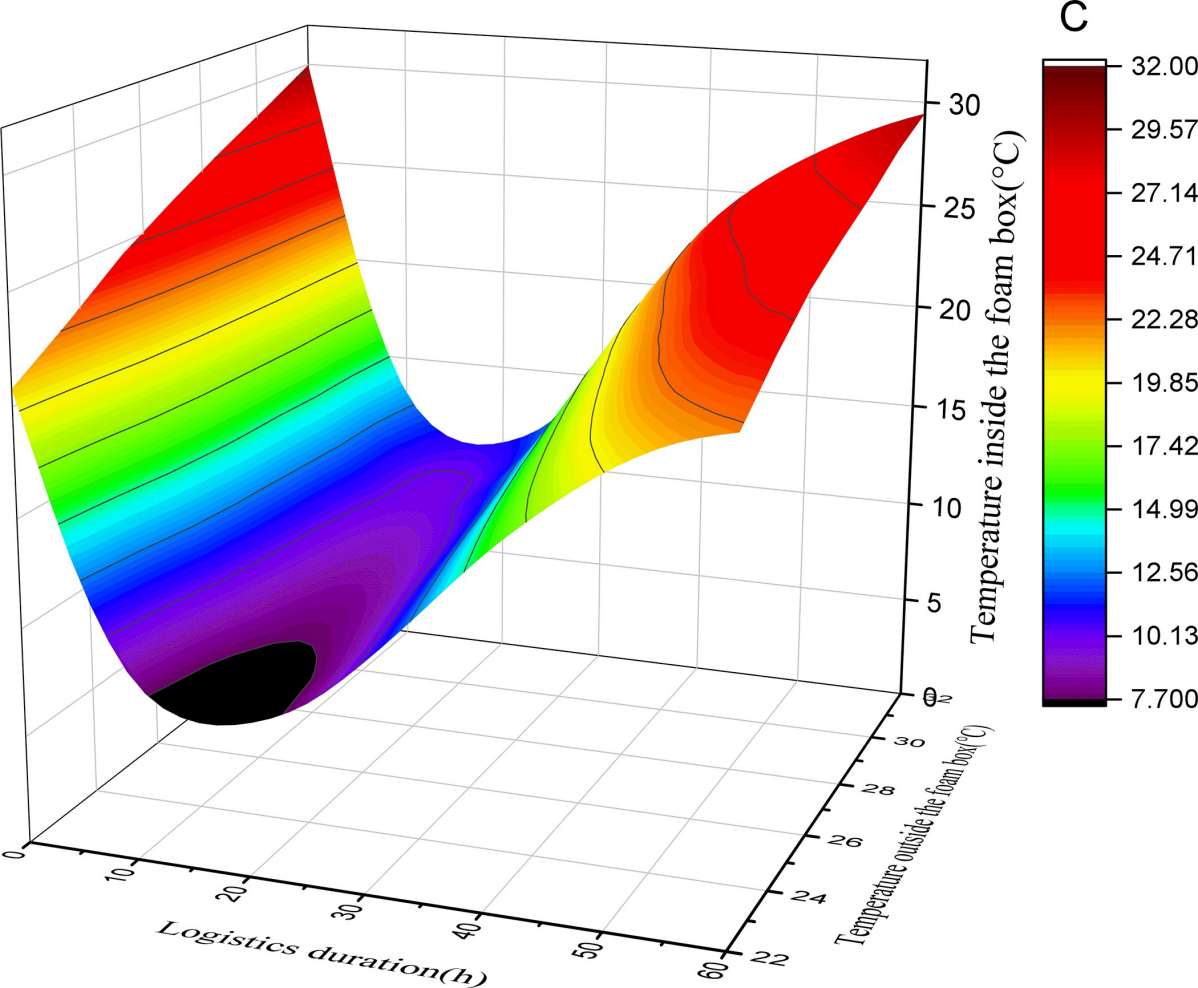
Surface diagram of temperature change in foam box (30% with ice).

**Fig 3:**
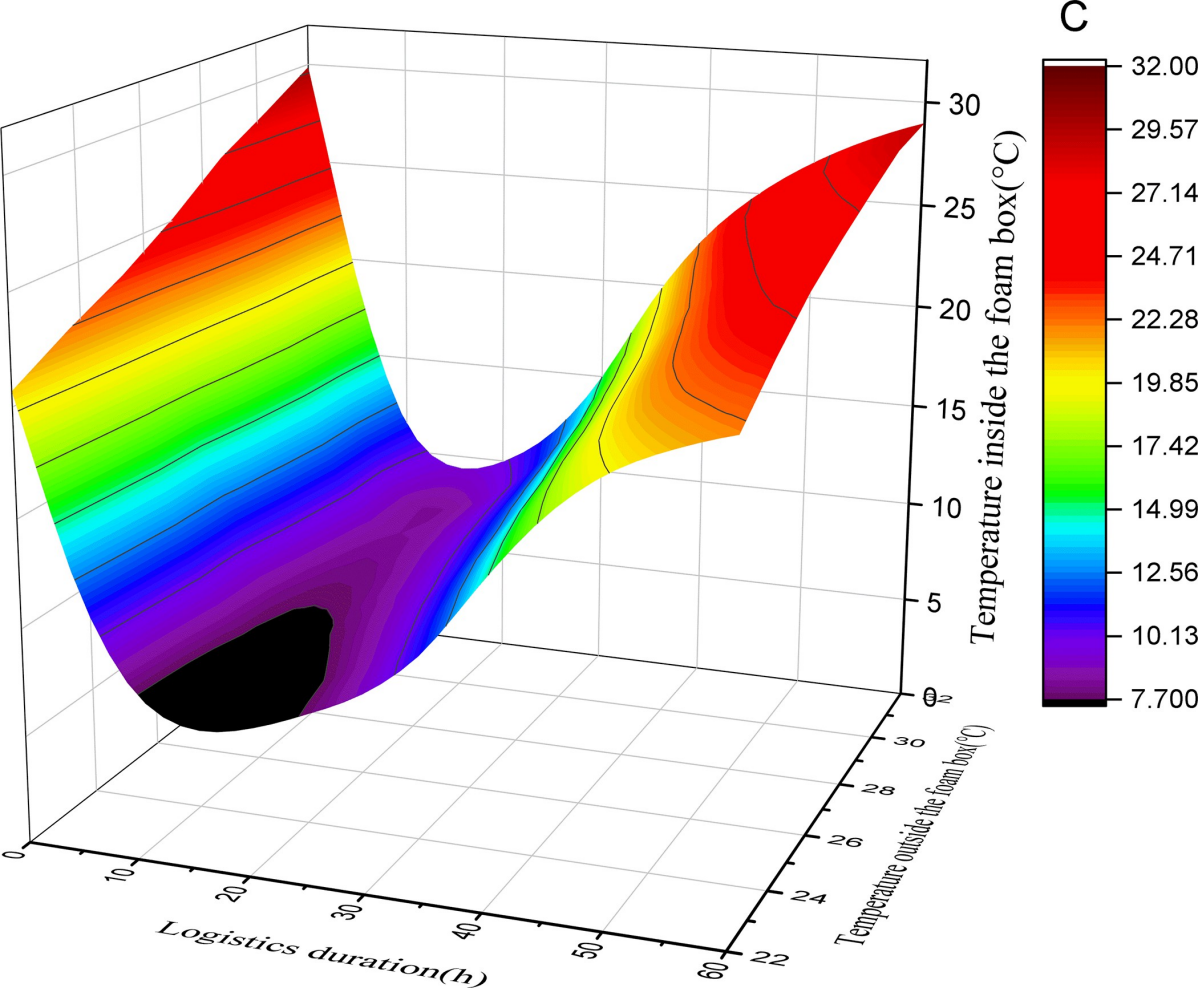
Surface diagram of temperature change in foam box (40% with ice).

**Fig 4:**
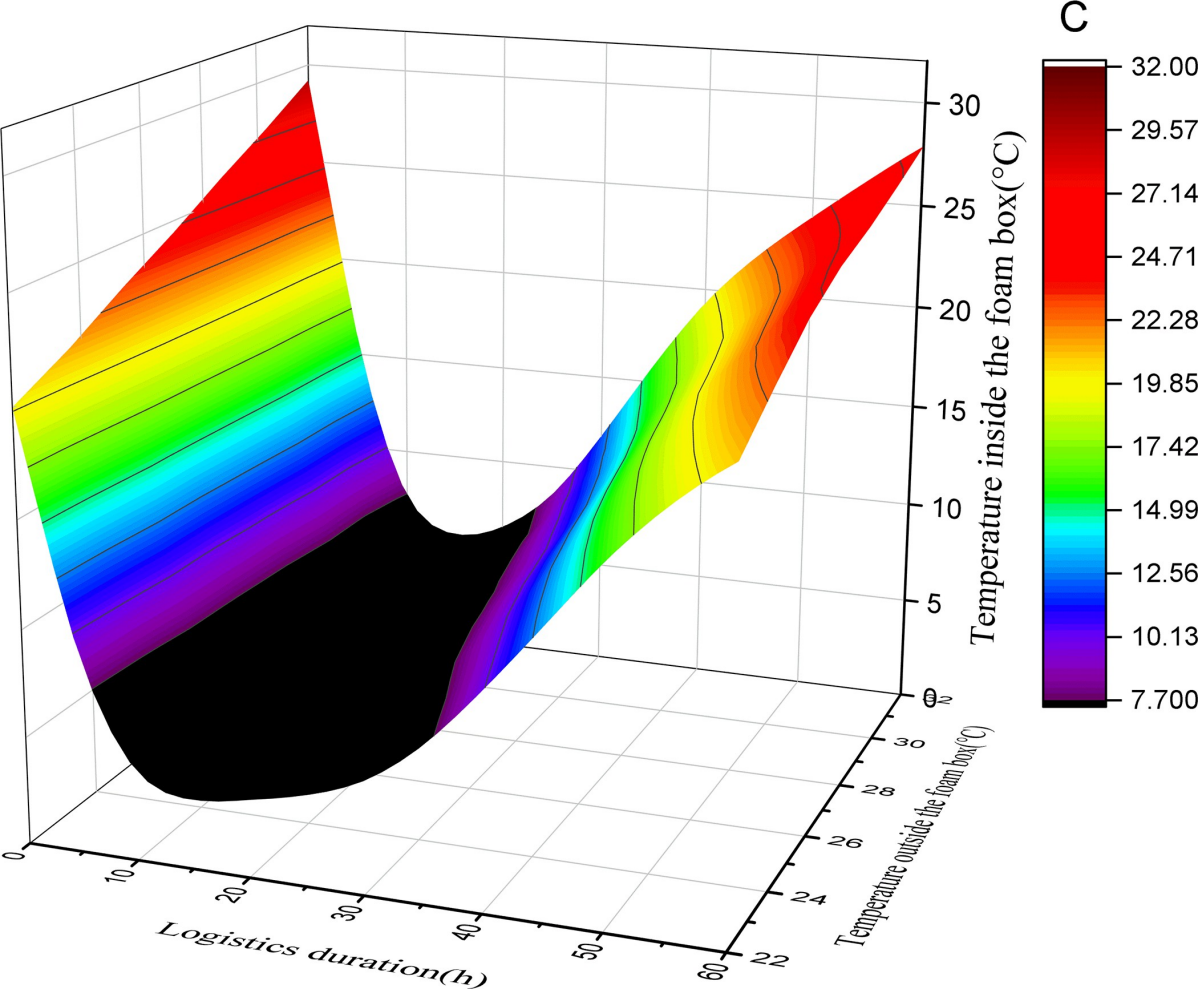
Surface diagram of temperature change in foam box (50% with ice).

This study assumes that the initial temperature of the foam box is equal to the outside temperature, the initial temperature of the pasteurized milk is 2°C, and the initial temperature of the ice added in the foam box is 0°C. By substituting the above data into Formulas (1), (4), (5) and (6), the relationship between the logistics duration, the external environment temperature and the temperature in the box corresponding to the different amount of ice added (50%, 40%, 30%, 20%) in the air pasteurized milk mode with foam box and ice can be obtained. See Figs 1–4.

According to the above calculation and observation results, the following rules can be found:

(1) The greater the amount of ice added, the lower the minimum temperature that can be reached in the foam box. When the amount of ice is 50% and the average external temperature is 22°C, the minimum temperature in the foam box can reach 3.9°C; With the change of external temperature, the minimum temperature that can be reached in the foam box will rise. When the average external temperature is 32°C, the minimum temperature that can be reached in the foam box is 5.2°C. With the addition of ice reduced to 20%, the lowest temperature that can be reached in the foam box increases. When the average external temperature is 22°C, the lowest temperature in the foam box can only reach 7.6°C. When the average outside temperature is 32°C, the lowest temperature that can be reached in the foam box is 13.3°C.

(2) The greater the amount of ice added, the faster the temperature in the box drops. As reflected in the figure, when the amount of ice added is 50% and the average external temperature is 22°C, the temperature change curve is the steepest and the absolute value of the slope is the largest; while when the amount of ice added is 20% and the average external temperature is 32°C, the temperature change curve is the gentlest and the absolute value of the slope is the smallest. It can be seen that if you want to quickly obtain a certain low temperature, you need to increase the amount of ice.

(3) The greater the amount of ice added, the longer the duration of the foam box at a relatively low temperature; According to the basic principle of conservation of energy, when the foam box reaches the lowest temperature, the cooling capacity and cooling consumption will maintain a balance for a period of time, and the time that can be maintained is related to the amount of ice added, the more ice added, the longer the low temperature can be maintained, otherwise the shorter. According to the calculation and figure, when the amount of ice added is 50%, there is a low temperature period lasting for a long time in the box, and with the reduction of the amount of ice added, the low temperature period that can be maintained in the box is gradually reduced. In the case of the same amount of ice, the low temperature time that the foam box can maintain is shortened with the increase of the external average temperature.

(4) In the case of different amounts of ice added to the foam box, the temperature in the box will first drop, and then rise, the overall appearance of irregular U shape, it can be inferred that the final temperature in the box will be the same as the external temperature; However, the greater the amount of ice added, the longer the time point at which the temperature in the box is consistent with the outside temperature. The calculated results and figure show that under the condition of 50% ice addition, even if the average temperature of the outside is as high as 32°C, the temperature in the foam box is always lower than the outside temperature within 60 hours; In the case of adding 20% ice, the temperature in the foam box is consistent with the external environment temperature in less than 40 hours.

(5) In addition to the amount of ice added, the external environment temperature has a great influence on the temperature change in the foam box. As the average temperature of the external environment changes from 22°C to 32°C, it is necessary to increase the amount of ice to ensure a certain appropriate temperature in the foam box. Of course, as the average temperature of the external environment decreases, less ice can be added to maintain a certain appropriate temperature in the foam box.

### Effects of temperature and duration in the box on physicochemical indexes and flavor of pasteurized milk

The physical and chemical indexes of pasteurized milk are highly related to the refrigeration temperature, and the physical and chemical indexes directly affect the taste and shelf life, so the influence of temperature change on the physical and chemical indexes and taste must be considered when discussing the appropriate amount of ice [20]. According to the current research conclusion, the acidity, pH value, taste and tissue status of pasteurized milk can remain stable within 60 hours at 4°C refrigeration temperature, and the activity of bacterial protease and lipase has no significant change, which can be considered as no change in taste [21]. With the increase of refrigerated temperature, the time for the physical and chemical indexes to maintain stability and taste unchanged is shortened. Under the condition of 25°C, the time of maintaining the stable state of taste and tissue is shortened to 5h, and slight fat floating occurs between 6-13h, which is acceptable [22]. Between 13 and 22 hours of fat floating more, but still acceptable; After 23h, the physical and chemical indexes were completely unacceptable. Under the condition of 30°C, the time of maintaining the stable state of taste and tissue is shortened to 3h, and slight fat floating occurs between 4 and 7h, which is acceptable. Between 8 and 10h, more fat floats up, but it is still acceptable. After 11h, the physical and chemical indexes were completely unacceptable [23].

## Discussion and conclusion

Based on the above analysis, this paper recommends the following data for adding ice: If 50% of the ice is added, the transportation quality can be satisfied within 60 hours at all normal external temperatures; If the logistics duration is less than or equal to 24 hours, it is recommended to add about 20% of the ice; The logistics duration is about 36 hours, it is recommended to add about 30% ice; The logistics duration is about 48 hours, it is recommended to add no more than 40% ice; In the actual operation, it can be combined with the season and month, query the ambient temperature (starting place, destination), determine the average external temperature, and increase or decrease the proportion of ice adding according to the above data, so as to determine the more accurate ice adding data. In the case of determining the amount of ice added, combined with the average temperature during transportation, the maximum allowable time of logistics can be queried.

It should be pointed out that there are many factors affecting the change of the temperature in the foam box under the ice-adding mode. The actual aviation cold chain logistics operation often does not meet the theoretical model, but due to financial constraints, this paper does not deeply discuss the influence of humidity, solar radiation and wind on heat transfer. In addition, the purity, size and shape of the ice used in this study will also affect its melting rate and cooling capacity; Different types of pasteurized dairy products have different specific heat capacities and thermal characteristics, which may affect the results; Only foam box material (EPS) materials were considered in this study, and the fact that different materials have different insulation properties may affect the results. This study is a static theoretical derivation, limited by the complexity of the study, and does not consider various accidental factors in the actual logistics process. Therefore, the research results are for reference only. In the future research, further in-depth research is needed.

## Supporting information

S1 File (ZIP)
